# Inclusive Early Childhood Education for Children With and Without Autism: Progress, Barriers, and Future Directions

**DOI:** 10.3389/fpsyt.2021.754648

**Published:** 2021-10-29

**Authors:** Michael Siller, Lindee Morgan, Quentin Wedderburn, Sally Fuhrmeister, Asha Rudrabhatla

**Affiliations:** ^1^Marcus Autism Center, Children's Healthcare of Atlanta, Atlanta, GA, United States; ^2^Department of Pediatrics, Emory University School of Medicine, Atlanta, GA, United States

**Keywords:** autism, inclusion, preschool, early childhood education, early intervention

## Abstract

University-affiliated lab and model schools play an important role in creating educational innovations in inclusive early childhood education (ECE) for young children with Autism Spectrum Disorder (ASD). In the United States, access to inclusive high-quality ECE programs for young children with disabilities has been required by law for over 40 years, has been recommended by leading professional organizations, and has been emphasized in federal public policy initiatives. Yet, improvement in the rates of young children with disabilities experiencing inclusion has been limited. This review article consists of three parts. First, we identify and describe four barriers to wide-scale implementation of inclusive ECE programs for children with ASD in the US. These barriers include (1) the fragmented nature of the ECE system in the United States, (2) the age at which ASD is typically first diagnosed in the community, (3) the diverse presentation/support needs of children with ASD, and (4) the thoughts and feelings of parents of children without disability about inclusion. Second, we used a snowball sampling approach to identify nine leading university-affiliated, inclusive lab and model schools for young children with ASD. By describing these programs, we highlight similarities and differences between programs, and capture the unique ways in which these programs adapt to local conditions, resources, and barriers (e.g., federal and state regulations, funding sources, community resources, institutional structures and priorities, professional orientation and training, access to families and staff). Finally, we propose a roadmap for researchers focused on the development, evaluation, and implementation of community-viable inclusive ECE programs in ASD. This roadmap leverages synergies between inclusive university-affiliated lab and model preschools in ASD, and proposes the formation of a research network that creates an infrastructure for cross-program collaboration.

## Introduction

Adopted in 2006, the United Nations Convention on the Rights of Persons with Disabilities requires that “States Parties … shall ensure that … persons with disabilities can access an inclusive, quality and free primary education on an equal basis with others in the communities in which they live” [(1), Article 24]. Further, the United Nations Educational, Scientific and Cultural Organization (2) defines inclusion as a “process of addressing and responding to the diversity of needs of all learners through increasing participation in learning, cultures and communities, and reducing exclusion within and from education” (p. 13). Guided by the conviction that it is the responsibility of the regular system to educate all children, individual differences among students are viewed “not as problems to be fixed, but as opportunities for enriching learning” for all children (p. 9). Moreover, while the UN Convention on the Rights of Persons with Disabilities focuses on primary education, international organizations such as the Enabling Education Network (https://www.eenet.org.uk) have highlighted opportunities for inclusion within Early Childhood Education (ECE) settings. Due to its focus on foundational learning skills (e.g., cooperating, personal skills like managing emotions, physical skills like manipulating small objects) and play-based learning, early childhood settings are ideally suited for promoting inclusive learning opportunities from early on (3).

Despite world-wide efforts to promote inclusive education, the origin and application of these efforts differs substantially by country and geographic region. For example, in northern countries (including the US), inclusion emerged as a response to segregation of students with disabilities in special education and mainstreamed settings, while developing countries tend to be more broadly concerned with school access for a wider range of children (4). While there is not a single model for promoting inclusion of children with disabilities that is applicable across the globe, important lessons can be learned from the journeys of individual countries. Guided by this approach, the current article focuses on the unique conditions, barriers, and opportunities for inclusive ECE in the United States. Further, because the current manuscript aims to develop a roadmap for researchers focused on the development, evaluation, and implementation of community-viable inclusive ECE options for children with ASD, our review explores the conditions of ECE inclusion in the US with a focus on this population of children.

### Access to Inclusive Early Childhood Education for Students With Disabilities in the US

In 2015 and 2017, the US Departments of Education (US DOE) and Health and Human Services (US DHHS) published a joint policy statement, stressing that “all young children with disabilities should have access to inclusive high-quality early childhood programs” (5). Access to inclusive learning environments has been required by US law for over 40 years [Individuals with Disabilities Education Act (IDEA), (6)], and strongly recommended by two prominent ECE organizations in the US [Division for Early Childhood (DEC) and National Association for the Education for Young Children (NAEYC), (7)]. Yet, improvement in the rates of children experiencing inclusion has been insubstantial (8, 9). Barton and Smith (10) used annual reports to congress prepared by the USDOE to estimate the percentage of children with disabilities, aged 3-5 years, who receive special education and related services in regular ECE classrooms. Although the classification terminology has changed somewhat across the decades, the authors estimate that this percentage increased from 36.8% in 1984/1985 (11) to 42.5% in 2011/2012 (12). Data from the most recent report to congress (12) indicate that this percentage continued to increase to 45.5% in 2017/2018. Thus, between 1985 and 2018 (33 years!), the practice of providing special education to children with disabilities, 3-5 years, in regular ECE settings appears to have increased by <10%.

### Contextually-Based Interventions for Young Children With ASD

Just like world-wide educational policy leaders are embracing the value and practice of inclusion, the emerging consensus among intervention researchers in ASD has coalesced around the notion that, to the extent possible, learning opportunities for children with ASD should be embedded within children's natural environment, particularly within familiar daily life routines that are predictable, meaningful, motivating, and developmentally-appropriate [Naturalistic Developmental Behavioral Interventions, NDBI, (13)]. Relevant routines and interactions occur at home (e.g., caregiving activities, play, and common household tasks/chores), in the community (e.g., going to a store, visiting a park), and in settings where interactions occur with typically developing children (e.g., ECE classrooms).

The current focus on contextually-based interventions in ASD has several roots, both in science and society. First, research on behavioral learning techniques has shown that contingency-based skill building is most effective when it is embedded in social interactions and activities that are motivating, meaningful, and allow children to experience the natural contingencies of their own behavior (14). By teaching skills within children's natural environments with multiple materials and interactive partners, learning and generalization of skills is optimized (13). Second, clinical practice guidelines for young children with ASD emphasize the intensity of children's learning opportunities. It is commonly recommended that children with ASD spend at least 25 h per week actively engaged in planned learning activities (15). Embedding planned learning activities within and across natural environments provides a feasible strategy for maximizing the intensity of children's learning opportunities. Third, during the last decade, intervention researchers in ASD have begun to leverage implementation science methods to plan, adapt, and implement evidence-based practices in community settings (16, 17). Implementation science methods provide researchers with a new set of tools for (a) adapting intervention strategies to fit the settings in which they need to be implemented, and (b) partnering with community practitioners and systems that interact with young children with ASD (18). Finally, during the last decades, societal views on disabilities have undergone a paradigm shift, away from medical models that emphasize charity, treatment, and social protection, and toward social models that emphasize respect for difference, acceptance, participation, and inclusion (1). In addition to references to human rights and equity, advocates for inclusive education also emphasize its utility-related benefits, arguing that inclusion is potentially the most cost- and time-efficient way of improving access to education for all children (4).

## Barriers to Inclusive Early Childhood Education for Children with Autism Spectrum Disorder

Creating and sustaining inclusive ECE options for young children with ASD in US community settings is challenged by multiple factors, including (1) the fragmented nature of the broader ECE system in the United States, (2) the age at which ASD is typically first diagnosed in the community, (3) the diverse presentation/support needs of children with ASD, and (4) the thoughts and feelings of parents of children without disability about inclusion.

### The Fragmented Nature of the Broader ECE System in the United States

The historical roots of the ECE system in the United States can be traced back to two distinct streams, both emerging in the 1830s—day nurseries and nursery schools (19). Day nurseries emerged in response to pressures created by rapid industrialization and immigration and emphasized basic care and supervision. Nursery schools, on the other hand, emerged in the context of the educational reform movement, and envisioned ECE as a means of escaping the intergenerational transmission of poverty. Throughout the last 200 years, these two major functions (i.e., care and education) have remained separate, both in their own ways being shaped by large-scale, historical developments including: (1) the rise of workforce participation of women during the second half of the 20th century (20), (2) growing interest in school readiness (21), and (3) the “welfare reform” legislation of 1996, which included work requirements for poor women with young children. Given these conflicting values and historical forces, the broader ECE system in the US today varies greatly in terms of geography, public/private mix, and access/coverage.

Empirical data on the utilization of early childcare and education programs in the US must be gleaned from multiple data systems that are not fully integrated. Laughlin (22) evaluated data collected by the US Census Bureau during spring 2011, providing valuable information about childcare arrangements prior to children's 3rd birthday. Data indicate that childcare arrangements differed vastly, both by child age and maternal employment (for children <1 years and 1-2 years, 52 and 54% of mothers were employed, respectively). For employed mothers, 16% of children <1 year, and 30% of children 1-2 years attended an organized childcare facility (including day care centers, nurseries/preschools, and Early Head Start programs). For unemployed mothers, 3% of children <1 year, and 4% of children 1-2 years attended an organized childcare facility.

Annual data on the utilization of ECE programs of 3- and 4-year-old children in 2019 are reported by The National Institute for Early Education Research (23). In this report, the percentage of the population enrolled in ECE is reported separately, based on child age (3 and 4 years) and program type (i.e., Public Pre-K, Private ECE, Head Start). The presented data show that 35% of 3-year-olds, and 20% of 4-year-olds were enrolled in private ECE programs. In addition, 6% of 3-year-olds, and 37% of 4-year-olds were enrolled in public Pre-K (either state or locally funded). In 2019, 45 states (incl. D.C.) offered a state-funded preschool program, and programs differed vastly with regard to eligibility requirements (e.g., 33 state programs had an income requirement), access for 3-year-olds (offered by 32 states, including D.C.), the state agencies charged with primary oversight (81% of state preschool programs were administered at least partially by the State Education Agency), and state preschool policies related to program quality. Finally, in 2019, 7% of 3-year-olds, and 8% of 4-year-olds attended Head Start, a federally-funded, comprehensive early education program for low-income families. Because of the federal requirement that at least 10% of enrollment consists of children with disability, Head Start is a major provider of inclusive ECE services in the US.

The reports by Laughlin (22) and Friedman-Krauss et al. (23) paint a complex picture of the participation of US children in ECE programs. Access and coverage differ by age, the availability of public options, and the families' socio-economic circumstances. For public school systems, the provision of inclusive learning options for 3- and 4-year-olds typically requires accessing funding through the state-funded Pre-K or the federally-funded Head Start system. While Early Head Start provides center-based program options for children younger than three years, the number of funded slots is relatively low. According to the National (Early) Head Start Services Snapshots for 2018-2019, about 100,000 center-based slots were funded in Early Head Start (children <3 years), compared to about 650,000 slots in Head Start [children ≥ 3 years; (24, 25)]. Thus, creating inclusive learning options for children younger than 3 years requires the involvement of private ECE programs (e.g., day care centers, nurseries/preschools).

### The Age at Which ASD Is Typically First Diagnosed in the Community

During the last two decades, research has made tremendous progress with regard to early identification and diagnosis of ASD. As a result, in many cases, ASD can now be reliably diagnosed between 18 and 24 months of age (26). Advances in best practices related to early identification are reflected in a 2006 policy statement published by the American Academy of Pediatrics (27), asserting that Primary Care Providers (e.g., family physicians, pediatricians) administer formal screening tests during every well-child visit scheduled at 18 and 24 months, independent of known risk factors or reported concerns. Moreover, Primary Care Providers are urged to promptly refer children for Early Intervention services as soon as ASD is seriously considered as a possibility for diagnosis.

While the age of first diagnosis has gradually decreased during the last two decades, population-based studies reveal that most children with ASD in the US continue to be diagnosed after their 4th birthday (28). According to the most recent report by the Autism and Developmental Disabilities Monitoring (ADDM) Network, an active surveillance program that estimates the prevalence of ASD among children aged 8 years residing in 11 ADDM Network sites in the US, 18.5 out of 1,000 children meet surveillance criteria for ASD (1 out of 54 children), and 13.2 out of 1,000 children have a documented clinical ASD diagnosis (28). Among the children with a documented clinical ASD diagnosis, children's median age at first diagnosis was 51 months. The median age of children's first comprehensive developmental evaluation was 40 months, with 44% being first evaluated at or prior to 36 months, and 37% being first evaluated later than 48 months.

The intersection between (1) the complex ECE system in the US, and (2) characteristic delays in ASD diagnoses has important implications for children's access to inclusive ECE environments. On one hand, many children who are eventually diagnosed with ASD are enrolled in ECE programs prior to receiving a formal diagnosis. Thus, ECE teachers serve as an important source of social and professional support during a time when parents begin to recognize concerns about their child's social-communication development, navigate the diagnostic process, and begin to access ASD-specific resources. In many instances, ECE teachers begin to implement individualized instruction and classroom adaptations prior to children's ASD diagnoses. On the other hand, because most children with ASD do not receive their formal ASD diagnosis until they are 4 years of age or older, the Pre-K and kindergarten years are often the first realistic opportunity for implementing formal special education services in inclusive classroom settings.

### The Diverse Presentation/Support Needs of Children With ASD

While access to inclusive ECE placements is important, learning occurs when children are active, independent participants within their classroom communities. Thus, successful inclusion of children with ASD requires that educators provide adequate individualized interventions (by embedding instruction within/across routines, activities, environments) and classroom adaptations (by embedding organizational, communication, sensory, or behavioral supports to make content accessible) to ensure that children are actively engaged in learning throughout the preschool day (29). Consistent with current principles of developmentally-appropriate practice for all young children (30), a comprehensive understanding of active engagement goes beyond simple “task attendance” and emphasizes children's social emotional engagement, which is critical for learning in ASD (31–33). To date, only three rigorous intervention studies in ASD have used mediation analyses to investigate the intervention mechanisms underlying children's learning outcomes (33–35). While only one of these three studies was completed in the classroom context (33), all three studies reveal that treatment-related outcomes were mediated by children's social engagement with a supportive adult (e.g., child initiations, parent synchronous responsiveness and mirrored pacing, joint engagement).

The nature and intensity of individualized interventions and classroom adaptations necessary to maximize classroom active engagement in ASD varies greatly across children (29). Inclusive model and lab preschools for ASD implement one of two broad strategies to accommodate this variability in children's clinical presentation and support needs. Most programs are designed to maximize flexibility in accessing resources and supports. This includes (1) hybrid programs that integrate clinical/behavioral intervention services (e.g., funded through health insurance providers) and inclusive ECE programming, (2) ECE programs that are operated by local school systems and are able to access system-wide supports for children with disabilities (i.e., Individualized Education Programs), or (3) programs that are affiliated with academic training programs for teachers and related professionals, providing flexibility in classroom staffing due to the availability of student interns. Alternatively, inclusive model programs have elected to limit variability in clinical presentation and support needs as part of the enrollment process. That is, programs set and implement specific enrollment criteria to ensure that all children who attend the program are likely to be successful, given the program's existing teacher-student ratios and teacher qualifications. While procedures to limit eligibility seem inconsistent with philosophical aspirations of inclusive education, a certain level of screening seems necessary to ensure the community-viability of inclusive ECE options in the US. In fact, students with ASD enrolled in most inclusion programs are not representative of the population of children with ASD, either because children's educational needs are specified in their Individualized Education Programs, because parents select programs that are likely to meet their children's needs, or because programs/parents dismiss/withdraw children if their educational needs are not met (36).

### The Thoughts and Feelings of Parents of Children Without Disability About Inclusion

Wide-scale access to inclusive learning environments for children with disabilities can only become a reality when parents of typically developing children value the benefit of such experiences for their own children and for society, and eventually select inclusive over non-inclusive alternatives when making decisions about preschool enrollment. Because the early childhood period is critical for children's language and social development, parents tend to carefully weigh their options before making these important decisions. Research using surveys and qualitative interviews reveals that most parents of young typically developing children express general positive attitudes about the value and benefits of inclusive classrooms (37). When asked about possible benefits for their own children, parents of typically developing children emphasize social emotional outcomes (e.g., promoting acceptance and empathy), while benefits for their child's academic outcomes are expressed to a lesser extent (38, 39).

Research investigating attitudes about inclusion has also shown that parental attitudes differ based on the specific diagnosis of children to be included (40, 41). That is, more positive attitudes are expressed toward inclusion of children with hearing impairment, while inclusion of children with complex behavioral disorders including ASD are viewed more cautiously. Specific parental concerns include the potential for behavioral disruptions, teachers' ability to divide attention among all children (38, 39, 42), and whether professional preparation of the ECE workforce is adequate for meeting the needs of children with disabilities (43).

Most available research used survey- or interview-based research methods to investigate parental attitudes about inclusion. The interpretation of this body of literature is complicated by (1) concerns about the social acceptability bias inherent in survey-based research (44), (2) evidence suggesting that parents have limited knowledge of what childhood inclusion entails in practice (45), and (3) questions about the extent to which generalized attitudes about inclusion have direct implications for parents' enrollment choices for their children (42). Moreover, the exact mechanisms that explain individual variation in parental attitudes about inclusion are poorly understood. A better understanding of variables that give rise to or impact parental attitudes could guide future efforts to raise awareness about the benefits of preschool inclusion. The mechanisms that underlie parental attitudes about inclusion are likely complex and may include cultural [e.g., collectivistic vs. individualistic values, (45)], philosophical [e.g., whether social justice orientation may serve as a motivating factor, (46)], curricular [e.g., whether parents value socialization-related or academic outcomes for young children, (38)], or personal factors [e.g., personality traits such as parental conscientiousness, (40)]. Further, parental attitudes about inclusion have been linked to the amount and quality of the parents' prior experiences with individuals with disabilities and/or inclusive education (37, 39).

## Inclusive Laboratory and Model Preschools in ASD

For several decades, university-affiliated lab and model programs have played an important role in creating educational innovations in the area of ECE inclusion for ASD. Most programs originate at a specific time and place, and find unique ways of adapting to their local environment: (1) federal legislation and funding sources, (2) state-specific regulations, support structures, and funding mechanisms, (3) operating procedures and resources at the academic host institutions, (4) professional background and experiences of the program developers, (5) diagnostic and intervention resources in the community, and (6) access to teaching/intervention staff and student populations. While these unique adaptations are an important source of innovation, the uniqueness of each program also poses challenges for rigorous program evaluations (e.g., generalizability of results) and complicates efforts of program replication (e.g., community-viability). In the following, we will identify and compare existing university-affiliated lab and model programs in ASD with the goal of creating a roadmap for researchers focused on the development, evaluation, and implementation of community-viable inclusive ECE programs in ASD. By identifying and describing existing programs, we aim to identifying synergy and opportunities for research collaborations between these programs.

To identify programs eligible to be included in this review, we used a “snowball sampling” approach. That is, we identified an initial set of programs based on a review of the literature, and then contacted program directors with the request to nominate additional programs. Criteria to be included in this review were (1) the program is affiliated with an academic institution, (2) the program provides inclusive ECE experiences for children younger than 5 years, and (3) the program is specifically designed to address the learning needs of children with ASD. We opted for this “snowball sampling” approach over a systematic review of the published literature since our focus was not on integrating findings about student outcomes, but rather on identifying active, university-affiliated lab/model programs. Based on this sampling approach, we identified nine leading programs, all except one located within the US. The nine programs vary widely in how long they have been operating, how far they have advanced on their path toward creating a community-viable inclusion model, and the extent to which student outcomes have been evaluated empirically.

### Project DATA (Developmentally Appropriate Treatment for Autism) [University of Washington]

Project DATA is one of five programs within the Experimental Education Unit at the University of Washington Haring Center of Inclusive Education. The Haring Center first opened in 1964 as a pilot school for children with neurological injuries, and has since evolved and expanded to educate children with diverse backgrounds and needs. Since its inception in 1997, Project DATA has been the site of critical intervention/education research in ASD. With funding from a Model Demonstration Grant (U.S. Department of Education, Office of Special Education Programs), Dr. Ilene Schwartz and her colleagues set out to develop a program that is data-based, effective, developmentally-appropriate, and acceptable to consumers (47). Core components of Project DATA include (1) an inclusive early childhood experience (about 12 h per week), (2) extended intensive instruction (10-12 h per week), (3) technical and social support for families (e.g., assistance with transportation, etc.), (4) collaboration and coordination across systems of childhood service, and (5) a quality-of-life influenced curriculum. During the inclusive early childhood experience, six children with ASD and 10 typically developing children are supported by 3-4 staff members. Extended intensive instruction is implemented in three sessions of eight children with ASD each. Students in each session are supported by 4-6 staff members, led by a Board Certified Behavior Analyst (BCBA) and assisted by masters students in applied behavior analysis (ABA). Currently, Project DATA is funded through grants from local school systems. Preliminary data from quasi-experimental research suggest that children who complete the program show significant developmental gains in adaptive (+22%), cognitive (+11%), social communication (21%), social (+24%), and fine motor (+30%) domains over the span of 16 months (47).

### Early Emory Center for Childhood Development and Enrichment [Emory University]

Early Emory was founded as the Walden Learning Center at the University of Massachusetts at Amherst in 1985 and moved to Emory University in 1991. Currently, Early Emory operates four age-grouped classrooms (64 children total, including 21 children with ASD), starting from Toddler (1-year-olds) to Pre-K (4-year-olds). Early Emory is guided by principles of incidental teaching and ABA, implements at least 30 h of instruction per week, and emphasizes peer engagement, social interaction, and parent involvement (48). The Early Emory curriculum employs the use of “teacher zones” in the classroom; teachers are trained to rotate across different “teacher zones” such that they develop the skills to engage children across different classroom activities and routines. Classroom-based ABA treatment and family engagement are integral components of the program. Parents are extensively coached for the first 6 months of their child's enrollment, biweekly for the next 6 months, and monthly for the remaining sessions. As a part of the childhood enrichment program, families are also encouraged to participate in monthly family activities and bi-annual parent teacher conferences.

Outcome data from Walden/Early Emory suggests three primary areas of improvement for children graduating from the program: verbalizations, peer interactions, and future placements. In a sample of 34 graduates, 30 children acquired meaningful verbal language (as defined by more than 10 verbalizations in functional, unprompted speech) with a 10% increase in verbalizations on average (49). In terms of peer interactions, 17 of the 34 children who graduated from the program were receiving increased peer social bids relative to program entry (*M* = 11%, *Range*: 1–27%). Last, 26 out of the 34 Early Emory graduates enrolled in regular kindergarten programs with varying degrees of individualized supports.

### Alexa's PLAYC [Rady Children's Hospital-San Diego]

Alexa's PLAYC, formerly known as the Children's Toddler School (CTS), is lab-based ECE program at Rady Children's Hospital in San Diego. The program opened in 1998, serving eight children with ASD and eight typically developing children from 18 months to 3 years. Four children with ASD attend a morning session, and four children with ASD attend an afternoon session. CTS began as a partial replication of the Walden Program, including core program features such as comprehensive teacher training, ABA, incidental teaching, and parent training, with a classroom staffed by three teachers (50). However, CTS differs from the Walden program in the provision of 1:1 programming outside of the classroom (as opposed to classroom-based ABA), a broader range of behavioral treatment strategies (e.g., pivotal response training, discrete trial training), and use of augmentative and alternative communication modalities. Further, to facilitate replication of the preschool model, CTS elected not to use Walden's characteristic “teacher zones” and rotation. CTS was re-named Alexa's PLAYC in 2010 when it expanded to include preschool in addition to toddler programs.

Using a quasi-experimental design, Stahmer and Ingersoll (50) reported on outcomes of 20 children with ASD served by CTS for a minimum of 6 months. Standardized assessments and measures of functional outcome were compared at program entry and exit. Results revealed significant increases in standard scores on measures of cognitive development and adaptive behavior as well as significant improvements in functional measures (e.g., response to others' initiations and engagement in reciprocal interaction). Compared to 11% at entry, 37% of the children were functioning in the typical range on measures of cognitive development at exit. A 10-year report of 102 children with ASD participating in the CTS yielded similar findings with significant improvements observed in developmental level, adaptive behavior and communication after an average of 8 months of program participation (51).

### Achievements [Kennedy Krieger Institute]

The Achievements program is a lab-based ECE program operated at the Center for Autism and Related Disorders (CARD) at the Kennedy Krieger Institute in Baltimore, Maryland. Founded by Dr. Rebecca Landa in the early 1990s, Achievements offers a variety of clinical models for children, from 22 months to 6 years of age. The program has eleven classrooms across two locations with a total capacity of 46 children. Each classroom serves 3–5 children; children are supported by one speech-language pathologist and 1–2 therapeutic assistants per class. Students also receive occupational therapy once per week and psychology and social work consults as needed. Attendance is billed through insurance as group therapy. Typically developing children from the Model Inclusion Childcare Classroom at CARD participate in the classroom as peer models. Instructional strategies used in the classroom include a continuum of approaches ranging from highly structured to routines-based intervention approaches. Visually-based organizational systems are provided and augmentative and alternative communication systems are used as needed. Achievements specifically targets socially engaged imitation, joint attention, and affect sharing.

Outcome data have been reported for the Early Achievements (for 2-year-olds) program in two published randomized trials (RCTs). Landa et al. (52) evaluated the impact of supplementing a comprehensive intervention (i.e., the early childhood program at Kennedy Krieger) with a curriculum targeting socially synchronous behavior [later referred to as Early Achievements; (53)]. For this study, 50 toddlers with ASD (aged 21–33 months) were randomized to either the comprehensive classroom intervention alone or the classroom intervention plus the Interpersonal Synchrony curriculum. Socially engaged imitation more than doubled with a significant treatment effect in favor of the Interpersonal Synchrony group. Imitation skills generalized to unfamiliar contexts and were maintained through follow-up. Similar gains were observed for initiation of joint attention and shared positive affect, but between-group differences did not reach statistical significance.

More recently, Early Achievements has been translated and tested in public childcare settings (53). Forty-eight childcare providers from 27 centers and 46 toddlers with social and/or communication delays (mean age = 28.5 months) participated in a cluster-randomized controlled trial. Early Achievements was adapted to community settings (Early Achievements for Childcare Providers; EA-CP) and compared to instruction-as-usual. EA-CP is delivered within shared book reading activities and includes a coaching framework that is implemented over 5-months and targets the use of various NDBI strategies (13). At the end of the study, providers in the EA-CP condition were implementing the intervention at an average of 80% fidelity. Although students in the EA-CP condition did not demonstrate significantly greater scores on developmental assessments than those in the instruction-as-usual condition, they did demonstrate significantly greater change in raw scores (*M* = 4.4, SD = 4.6) on the Social-communication Assessment in Book Sharing [SABS, (54)] compared to toddlers in control classrooms with a large effect size in favor of the EA-CP group.

### Preschool Education Lab [Marcus Autism Center/Emory University]

The Preschool Education Lab (PEL) at Marcus Autism Center/Emory University opened in 2018 and was developed by Dr. Michael Siller and Dr. Lindee Morgan. PEL functions as model inclusion preschool and a as a laboratory preschool to advance the science of inclusive ECE in ASD. Community viability is central to the program's design. That is, the program is designed to operate under the same financial and operational constraints as comparable high-quality preschool programs in the community. PEL is a full-day preschool program that is licensed as a Child Care Learning Center by the state, participates in state-wide quality improvement processes (Quality Rated Child Care), and implements state-wide early learning standards. The program includes two tuition-funded classrooms for 2- and 3-year-olds, and a state-funded Pre-K classroom for 4-year-olds. The three classrooms include 12, 16, and 18 children, respectively. Each classroom includes six children with ASD and is supported by a team of three teachers. All teachers have degrees/experiences in early childhood education (at the BA or AA level), but no specialized training in ASD interventions (with the exception of the Pre-K classroom which includes one teacher with a special education background). The teaching staff is supported by a classroom coach (30% effort) who supports the teachers in developing individualized learning outcomes and classroom supports. PEL uses the SCERTS framework (55) to develop individualized student outcomes and tailor classroom supports for students with ASD. Direct 1:1 intervention sessions are not implemented as part of PEL, although some children may be supported by community-based speech-language or ABA therapists during part of the day.

Because the general program structure (e.g., teacher-student ratio) of PEL is relatively fixed and the ability to allocate additional individualized resources is limited, the program has developed an eligibility process to ensure that all enrolled students with ASD are ready for the provided classroom-based learning experiences. The eligibility process includes a combination of parent surveys, a structured eligibility observation with a clinician/researcher, and a classroom visit [a detailed description of eligibility and enrollment procedures is reported in (36)]. Program outcome data have not been published to date.

### Early Learning Institute [Michigan State University]

The Early Learning Institute (ELI) at Michigan State University was developed by Dr. Joshua Plavnick (BCBA-D) and Laurie Linscott (M.A.) in 2015. The program is housed within the Child Development Laboratories and was created with the goal of providing early intervention services to children with ASD and creating a context for training service providers to learn about evidence-based practices. ELI utilizes a combination of ABA and parent coaching to address the needs of families. To be eligible, children must be diagnosed with ASD, be between the ages of 2 and 4 years by the beginning of the program, and be eligible for high level of ABA services. Once enrolled, children attend the program from 8:30 AM to 4:00 PM Monday through Thursday, year-round. Although an ASD diagnosis is a prerequisite for enrollment, there are opportunities to foster inclusive settings within the broader Child Development Laboratories classrooms. To date, the program has served a total of 29 families and trained 19 researchers and service providers. As a relatively newer program, outcome data on children completing ELI have not been published.

### Susan Gray School [Vanderbilt University]

The Susan Gray School (SGS) is operated by Peabody College at Vanderbilt University, a world-class college of education and human development. Originally named the Peabody Experimental School, SGS opened in 1968 as an on-campus research-oriented school devoted to educational research involving young children with developmental disabilities and children whose future development was at risk because of conditions such as poverty. Currently, SGS offers eight classrooms from infancy through Pre-k, serving about 92 students in total (30% with disabilities; 10% from economically disadvantaged backgrounds). The school has 16 full-time teachers. Moreover, SGS functions as a training site for students from various disciplines including Special Education, Teaching and Learning, Psychology, Human Development, and Speech and Hearing. Aside from the eight inclusion classrooms, SGS also includes a community outreach program, which currently serves children with developmental delays/disabilities from birth to 36 months (about 20 case visits per week). While faculty affiliated with SGS have produced a range of publications on inclusive ECE, program outcome data have not been published to date.

### Learning Experiences and Alternative Program for Preschoolers and Their Parents

Learning Experiences and Alternative Program for Preschoolers and Their Parents (LEAP) is a manualized intervention approach that aims to enhance the social interactions of young children with special needs. LEAP can be implemented within high-quality, general education settings for preschool-aged children. Key features of LEAP include: (1) targeting of individualized objectives within classroom activities with an emphasis on peer-mediated methods, (2) systematic focus on generalization, and (3) achievement of intervention intensity by maximizing instructional opportunities (56).

Strain and Bovey (57) completed an RCT of LEAP in 56 inclusive classrooms serving 294 children with ASD. The program implementation included structured parent-training component and detailed treatment fidelity procedures. After a 2-year period of training and mentoring, children in LEAP classrooms showed significantly greater improvement than controls on measures of global development, language, social skills, behavior, and ASD symptoms (moderate to large effect sizes). Further, results from a long-term follow up study showed intervention-related gains in social and cognitive skills were maintained over a 4-year post intervention period (58). A recent cluster RCT comparing LEAP to TEACCH-based and non-model specific classrooms showed that all children, independent of treatment condition, showed comparable improvements after 1 year of intervention implementation (59).

### Group—Early Start Denver Model

Another example of a manualized intervention model for inclusive ECE classrooms is a group-based adaptation of the Early Start Denver Model [ESDM (60)]. ESDM was originally designed to be delivered by a trained therapist on a 1:1 basis with implementation targeted for 15-20 h per week. This approach has demonstrated positive effects on improving cognitive, adaptive, and language outcomes for young children with autism (61, 62). Group—Early Start Denver Model (G-ESDM) was developed to provide children with ASD intervention within the context of a high-quality ECE classrooms. The primary goals of the G-ESDM are to support active engagement in group activities and routines throughout the school day with an emphasis on promoting the use of communication with peers and adults, successfully negotiate transitions, and develop skills necessary for participation in classroom environments (63, 64). Individualized goals are addressed using a variety of intervention strategies consistent with NDBI (13), including antecedent-behavior-consequence contingencies, peer-mediated teaching, and strategies for emotional and motivational regulation.

To evaluate the feasibility and initial efficacy of implementing G-ESDM in inclusive settings, Vivanti et al. (65) randomized 44 preschoolers with autism to either inclusive or special education classrooms, all implementing G-ESDM. After 12 months, children in both classroom types showed equivalent and significant gains on proximal measures of spontaneous communication and social interaction as well as distal measures of verbal cognition, adaptive behavior, and autism symptoms.

## Leveraging Laboratory and Model Preschools to Create A Program of Research to Advance The Science of Inclusive Early Childhood Education in ASD

Successful lab or model preschools in ASD find unique ways of leveraging local resources, including (1) affiliations with academic training programs in education, ABA, and related disciplines to create training opportunities and improve teacher-student ratios (e.g., Susan Gray School, Project DATA, Early Learning Institute), (2) opportunities to create hybrid programs that combine intensive clinical/behavioral intervention services (e.g., ABA) with inclusive learning opportunities within ECE classrooms (e.g., Project DATA, Early Learning Institute, Early Emory, Alexa's PLAYC, Achievements), and (3) affiliations with local school systems (e.g., Project DATA, LEAP). As emphasized above, these unique adaptations are an important source of innovation. At the same time, the uniqueness of each program also poses challenges for rigorous program evaluations (e.g., generalizability of results), and complicates efforts of program replication (e.g., community-viability).

In the following, we will propose a roadmap for researchers focused on the development, evaluation, and implementation of community-viable inclusive ECE programs in ASD. This roadmap leverages synergies between inclusive university-affiliated lab and model preschools in ASD, and proposes the formation of a research network that creates an infrastructure for cross-program collaboration. During the last decades, similar research networks have led to significant advances in the science of early identification (i.e., Autism Baby Siblings Research Consortium; https://www.babysiblingsresearchconsortium.org/) and early intervention [i.e., Autism Speaks Toddler Treatment Network (66)] in ASD. The proposed research program includes nine interrelated research aims.

### Research Aim #1: Create a Comprehensive, Systematic Review of Existing Inclusive Laboratory/Model Preschool Programs in ASD

The current chapter provides brief descriptions of nine programs, identified through a snowball sampling approach. This approach is limited in several regards. First, the list of included programs is likely incomplete, particularly with regard to programs that are more loosely affiliated with academic institutions and programs that operate outside of the US. Second, the information used to describe individual programs was largely based on online searches and published literature. Future research should use a standardized data collection process (e.g., program director surveys or interviews) to gather information about similarities and differences between programs more systematically.

### Research Aim #2: Support Inclusive Laboratory/Model Preschool Programs in Publishing Outcome Data

Because of the nature of these programs, experimental research designs are often not feasible when evaluating learning outcomes of children enrolled in individual inclusive lab/model preschool programs (e.g., biased, self-selected samples, lack of adequate control groups, ethical/practical concerns about random assignment). However, given the current state of the field, carefully planned quasi-experimental designs and pre-post comparisons of enrolled students can provide useful information about (1) promising outcome measures, (2) measures that predict intervention response, and (3) process measures (e.g., classroom active engagement) that explain individual differences in learning outcomes. Most importantly, this research should focus equally on students with and without ASD. A better understanding of the benefits of inclusive learning environments for typically developing children could inform parents' enrollment decisions as well as the programs' curricula and educational approaches.

### Research Aim #3: Facilitate Collaborations Between Inclusive Laboratory/Model Preschool Programs to Investigate Shared Process and Outcome Measures

To gain a mechanistic understanding of the processes that underlie learning in inclusive classroom environments, inclusive lab/model preschool programs should collaborate and collect shared process and outcome measures. The collection of process measures may involve a standard protocol for collecting classroom videos, and observational coding systems to capture elements of student active engagement (e.g., investment, independence, social initiations) and teacher measures of implementation fidelity (e.g., individualized interventions, classroom adaptations).

### Research Aim #4: Create an Implementation Science Framework for Scaling Existing Inclusive Laboratory/Model Preschool Programs in Community Settings

Most inclusive lab/model preschool programs have intermediate- or long-term plans to create a generalizable inclusion model that can be replicated and implemented in community settings. However, given the complexity of the ECE system in the US, the field would benefit greatly from a consistent framework for adapting, manualizing, replicating, and scaling inclusion models. Community implementation may either focus on scaling entire program or classroom models (e.g., Alexa's PLAYC), or focus on program components central to the inclusion model (e.g., LEAP).

### Research Aim #5: Use Causal/Experimental Methods to Evaluate the Learning Outcomes of Children With and Without ASD

Eventually, wide-scale implementation of inclusive preschool models for children with ASD will require rigorous research documenting program efficacy/effectiveness for both children with and without ASD. This research should involve collaborations between existing lab/model programs, focus equally on short-term and long-term child outcomes, resist temptations of “intervention branding,” and investigate learning outcomes associated with intervention mechanisms shared across different programs. The NDBI moniker may serve as a fruitful framework for this work.

### Research Aim #6: Investigate the Feasibility of Inclusive ECE Models Across Multiple ECE Systems

As described above, the ECE education system in the US is rather complex, and inclusion models will need to be adapted to meet the needs of different service systems, including public Pre-K, private childcare, Head Start, and Early Childhood Special Education. Moreover, because early identification and intervention are crucial components of effective intervention programs in ASD, the field requires inclusive ECE options for infants and toddlers, including children who have not yet received a formal ASD diagnosis. Such programs should find ways to leverage available early intervention (Part C) resources.

### Research Aim #7: Investigate Whether Inclusive Options Should Be Specific to ASD or Incorporate Children Across Multiple Societal or Disability Categories

Efforts to create an inclusive society need to move beyond targeted inclusion programs for specific societal categories (e.g., children with ASD), and strive toward learning environments where all young children are accepted and supported in accordance with their unique learning style and needs. However, this philosophical orientation may be at odds with the practical constraints inherent in effective and efficient workforce development. The field would benefit from comparisons between inclusion programs targeting ASD, and inclusion programs targeting children with disabilities/developmental delays more broadly. Importantly, this work should focus equally on student (e.g., development, learning) and teacher (e.g., efficacy, burnout) outcomes.

### Research Aim #8: Gain a Better Understanding of How Parents of Typically Developing Children Think and Feel About ECE Inclusion

Parents of typically developing children who seek enrollment in inclusive lab/model preschool programs constitute a highly self-selected group of families. To make inclusive ECE programs a reality on a larger scale, the field would greatly benefit from a better understanding of factors that influence parental thoughts, feelings, and buy-in. This information could inform individual programs' procedures for student recruitment as well as population-wide public awareness campaigns about the benefits of inclusive ECE.

### Research Aim #9: Develop Adaptive Interventions That Guide Decisions About Combining/Transitioning Between Clinician-Delivered Interventions and Classroom-Based Inclusive Learning Opportunities

It is likely that not all children with ASD benefit equally for inclusive, classroom-based learning environments. Some children may require intensive clinician-delivered interventions prior to transitioning to inclusive ECE programs. Other children may benefit from a combined approach that integrates clinician-delivered and classroom-based learning opportunities. Recent advances in the evaluation of adaptive interventions (67) provide a framework for embedding evidence-based decision points within children's comprehensive intervention/education programs.

## Conclusion

During the last decade, intervention researchers in ASD have converged on the notion that, to the extent possible, learning opportunities should be embedded within children's natural environment and involve (1) play or familiar daily life routines that are meaningful, rich in affect, and motivating, and (2) quality relationships with other people, including adults and peers (13). ECE programs provide a prime context for creating and embedding these kinds of learning opportunities for children with ASD. University-affiliated model and laboratory schools play an important role in creating the educational innovations necessary to flexibly integrate clinical/behavioral interventions and inclusive ECE programs to meet the learning needs of young children with ASD. Further, collaboration between these university-affiliated model and laboratory schools has the potential to impact community practice by establishing consensus about the essential elements of high-quality inclusion, developing a shared measurement framework for program fidelity and child outcomes, collaborating on large-scale effectiveness trials, and creating an implementation framework for moving educational innovations from university-affiliated to community programs.

## Author Contributions

MS wrote the first draft and outline of the manuscript. MS, LM, QW, and AR wrote sections of the manuscript. All authors contributed to manuscript revision, read, and approved the submitted version.

## Funding

This study received funding from The Marcus Foundation, Children's Research Trust, and Chesed Inc. The funders were not involved in the study design, collection, analysis, interpretation of data, the writing of this article or the decision to submit it for publication.

## Conflict of Interest

The authors declare that the research was conducted in the absence of any commercial or financial relationships that could be construed as a potential conflict of interest.

## Publisher's Note

All claims expressed in this article are solely those of the authors and do not necessarily represent those of their affiliated organizations, or those of the publisher, the editors and the reviewers. Any product that may be evaluated in this article, or claim that may be made by its manufacturer, is not guaranteed or endorsed by the publisher.
